# Supplementary material for: Ageing-Related Neurodegeneration and Cognitive Decline
Source: Int J Mol Sci. 2024 Apr 5;25(7):4065. doi: 10.3390/ijms25074065 (PMC11012171; doi:10.3390/ijms25074065)
Supplement: Supplementary file 1 [file ijms-25-04065-s001.zip › ijms-2904913-supplementary.pdf]

Supplement Table S1. Brain regions to be assessed and assessments used

Sections and stain's needed for assessment of pathological alterations.					
Organ and/or region	HE	IHC/HPτ	IHC/Aβ	IHC/pTDP43	IHC/pαS
Gyrus frontalis medius	X			X	X
Gyrus temporalis superior and medius	X	X		X	
Gyrus cingula	X				X
Gyrus parietalis inferior	X		X		X
Gyrus prae- & postcentralis	X				
Lobulus occipitalis	X	X			
Gyrus hippocampi/parahippocampi, anterior	X	X	X		
Gyrus hippocampi/parahippocampi, posterior	X			X	
Hypothalamus & amygdala & nucleus Meynert	X	X	X	X	X
Striatum	X				
Thalamus & nucleus subthalami	X				
Mesencephalon with substatia nigra	X		X		X
Pons with locus coeruleus	X	X			
Medulla oblongata with nucleus hypoglossus	X				X
Vermis cerebelli & nucleus dentatus	X		X		
Cortex cerebelli	X				

HE, hematoxylin-eosin; IHC, immunohistochemistry; HPτ, hyperphosphorylated τ; Aβ, β-amyloid; pTDP43, phosphorylated transactive DNA binding protein 43; pαS, phosphorylated α-synuclein.

Supplement Table S2. Immunohistochemistry

Antibody	Clone	Source	Dilution	Pre-treatment
Hyperphosphorylated τ (HPτ)	AT8	Thermo Scientific	1:500	-
β amyloid (Aβ)	6F/3D	Dako	1:100	80% Formic Acid, 6 hours
α synuclein (αS)	KM51	NovoCastra	1:100	Citrate Buffer pH 6.0* & 80% Formic Acid, 5min
transactive DNA binding protein 43 (TDP43)	11-9	Cosmo Bio	1:5000	Citrate Buffer pH 6.0*

* autoclave
